# Exploring T Cell Reactivity to Gliadin in Young Children with Newly Diagnosed Celiac Disease

**DOI:** 10.1155/2014/927190

**Published:** 2014-03-03

**Authors:** Edwin Liu, Kristen McDaniel, Stephanie Case, Liping Yu, Bernd Gerhartz, Nils Ostermann, Gabriela Fankhauser, Valerie Hungerford, Chao Zou, Marcel Luyten, Katherine J. Seidl, Aaron W. Michels

**Affiliations:** ^1^Barbara Davis Center for Childhood Diabetes, School of Medicine, University of Colorado Denver, Mail Stop A140, 1775 Aurora Court, Aurora, CO 80045, USA; ^2^Novartis Institutes for Biomedical Research, 4002 Basel, Switzerland

## Abstract

Class II major histocompatibility molecules confer disease risk in Celiac disease (CD) by presenting gliadin peptides to CD4 T cells in the small intestine. Deamidation of gliadin peptides by tissue transglutaminase creates immunogenic peptides presented by HLA-DQ2 and DQ8 molecules to activate proinflammatory CD4 T cells. Detecting gliadin specific T cell responses from the peripheral blood has been challenging due to low circulating frequencies and heterogeneity in response to gliadin epitopes. We investigated the peripheral T cell responses to alpha and gamma gliadin epitopes in young children with newly diagnosed and untreated CD. Using peptide/MHC recombinant protein constructs, we are able to robustly stimulate CD4 T cell clones previously derived from intestinal biopsies of CD patients. These recombinant proteins and a panel of *α*- and *γ*-gliadin peptides were used to assess T cell responses from the peripheral blood. Proliferation assays using peripheral blood mononuclear cells revealed more CD4 T cell responses to *α*-gliadin than *γ*-gliadin peptides with a single deamidated *α*-gliadin peptide able to identify 60% of CD children. We conclude that it is possible to detect T cell responses without a gluten challenge or in vitro stimulus other than antigen, when measuring proliferative responses.

## 1. Introduction

Celiac disease (CD) is a T cell mediated enteropathy triggered by the ingestion of dietary gluten resulting in villous atrophy and crypt hyperplasia in the small intestine [1]. Specific human leukocyte antigen (HLA) genes are involved in the disease process with restriction primarily limited to HLA-DQ2 (DQA∗05:01, DQB∗02:01 and DQA∗02:01, DQB∗02:02) and DQ8 (DQA∗03:01, DQB∗3:02) [2]. HLA genes encode class II major histocompatibility molecules (MHC), which present antigens to CD4 T cells. There has been remarkable progress in the understanding of the pathogenesis and epitopes involved in the disease process [3–6]. Gliadin, one of the two principle protein components of gluten, contains a number of well-studied T cell epitopes. Deamidation of gliadin peptides by tissue transglutaminase (TTG) type 2 converts glutamine into glutamic acid, resulting in immunogenic T cell epitopes [7].

Despite the current understanding of T cell—peptide—MHC interaction, simple and reliable T cell assays from the peripheral blood to monitor CD activity have been difficult to develop. Peripheral blood biomarkers for CD are hindered by variables such as the type of assay, use of the proper antigen, low circulating frequencies of T cells, and the timing of gluten exposure when the assay is performed. For example, it has been reported that, to obtain sufficient T cells for study without in vitro expansion, CD blood donors on a gluten-free diet need to undergo short-term gluten challenge, to detect IFN-*γ* T cell responses by enzyme linked immunospot (ELISPOT) assays [8, 9]. However, both gluten-free and gluten-exposed CD patients can have measurable T cell proliferative responses with response rates more frequent in gluten-exposed patients [10]. Comprehensive epitope mapping studies have identified four immunodominant DQ2 epitopes in treated CD adults (gluten-free diet) followed by a gluten challenge [11]. Such an unbiased epitope mapping project has not been undertaken for children with CD, even though there are several well-studied epitopes in the literature.

In this study, we investigated the peripheral T cell responses to alpha and gamma gliadin epitopes in young children with newly diagnosed and untreated CD. The selected epitopes are known to stimulate T cell clones derived from adult CD patient intestinal biopsies [12, 13]. Recombinant DQ2 and DQ8 proteins with *α*-gliadin epitopes were created to test T cell clone stimulation without the use of antigen presenting cells, as part of an effort to develop a T cell stimulation assay feasible for large scale, consistent, and rapid assessment of CD activity. We explored the utility of peptide/MHC complexes first in stimulating T cell clones and then peripheral blood mononuclear cells (PBMCs) of newly diagnosed CD children prior to treatment with a gluten-free diet to produce inflammatory cytokines. Subsequently, proliferation assays utilizing a panel of previously described DQ2 and DQ8 peptides on the same CD children provide insight into the possible degenerate nature of *α*-gliadin peptide binding motifs for HLA-DQ2.

## 2. Methods

### 2.1. Subjects and Samples

Subjects were recruited from the Children's Hospital Colorado, and written informed consent was obtained after the nature and possible consequences of the study were explained to individuals. The clinical investigation in this study was conducted in accordance with the Declaration of Helsinki principles, and study approval was provided by the Colorado Multiple Institutional Review Board. Peripheral blood was obtained for T cell assays, TTG antibodies, and HLA genotyping. TTG antibodies were measured from the serum by radioimmunoassay as previously described [14]. HLA-DRB, DQA, and DQB genotyping were performed using linear arrays of immobilized sequence-specific oligonucleotides similar to previously described methodology [15].

### 2.2. Expression and Purification of Recombinant Protein

**Figure 1:**
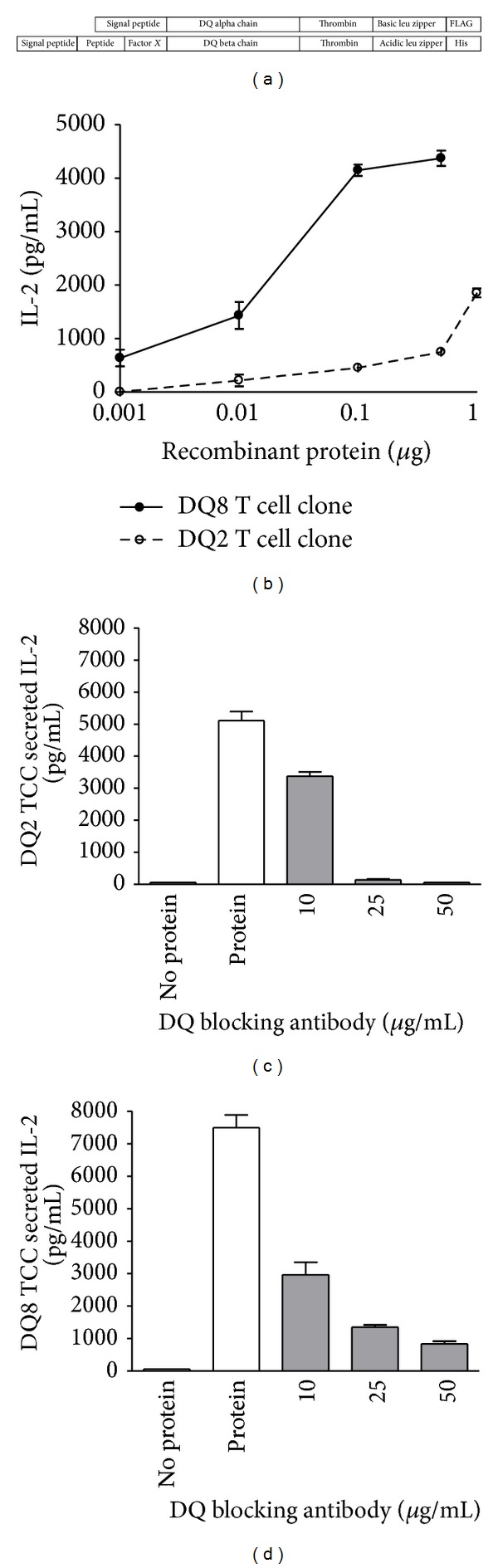
Recombinant DQ8 and DQ2 proteins with gliadin epitopes stimulate T cell clones. (a) Diagrams of the constructs used to produce recombinant protein for deamidated *α*-gliadin p1E, p9E/DQ8, and *α*-I gliadin/DQ2. The amino acid sequence of the *α*-gliadin peptide in DQ8 is QQYPSG**E**GSFQPSQ**E**NPQ and the *α*-I gliadin peptide (QLQPFPQP**E**LPY) with DQ2. Thrombin, TEV, and PreScission are protease cleavage sites incorporated into the protein constructs. (b) T cell clones restricted to either DQ8 or DQ2 produce IL-2 in response to the deamidated *α*-gliadin/DQ8 or *α*-I gliadin/DQ2 recombinant protein, respectively. (c) The DQ2 and (d) DQ8 T cell responses can be blocked in a dose dependent manner with a monoclonal DQ antibody.

The extracellular domains of the HLA-DQ8 *α* chain (residue 24–204) and *β* chain (residue 33–221) were coexpressed in S2 drosophila cells (ExpreS2ion Biotechnologies, Denmark). Double deamidated *α*-gliadin (QQYPSGEGSFQPSQENPQ) was covalently attached to the N-terminus of *β* chain with a Factor *X* cleavage site (GGGGSIEGRGSGGGS) between the peptide and the *β* chain. To stabilize the heterodimer, Fos and Jun leucine zippers were attached to the C-terminus of *α* chain and *β* chain via a thrombin cleavage sequence (SSADLVPRGS). Deamidated *α*-gliadin/DQ8 was extracted from the medium using anti-FLAG M2 (Sigma Aldrich) affinity chromatography. The recombinant protein was purified by Superdex 200 column in buffer containing 10 mMTris, pH 8.0, and 150 mM NaCl. For the *α*-I-gliadin/DQ2 construct, the extracellular domains of HLA-DQ2 *α* chain (residue 24–206) and *β* chain (residue 33–221) were used, expressed in S2 drosophila cells, and purified in a similar manner to the DQ8 recombinant protein construct. The amino acid sequence of *α*-I gliadin, QLQPFPQPELPY, was covalently attached to the N-terminus of the *β* chain via TEV cleavage site (GGGGENLYFQGGSGGGS). To stabilize the heterodimer, Fos and Jun leucine zippers were attached via PreScission cleavage site (SSADLEVLFQGP) to the C-termini of *α* chain and *β* chain, respectively. The final proteins were confirmed by LC-MS. Diagrams of the two recombinant protein constructs are depicted in Figure 1(a).

### 2.3. Generation of T Cell Receptor Hybridomas

**Figure 2:**
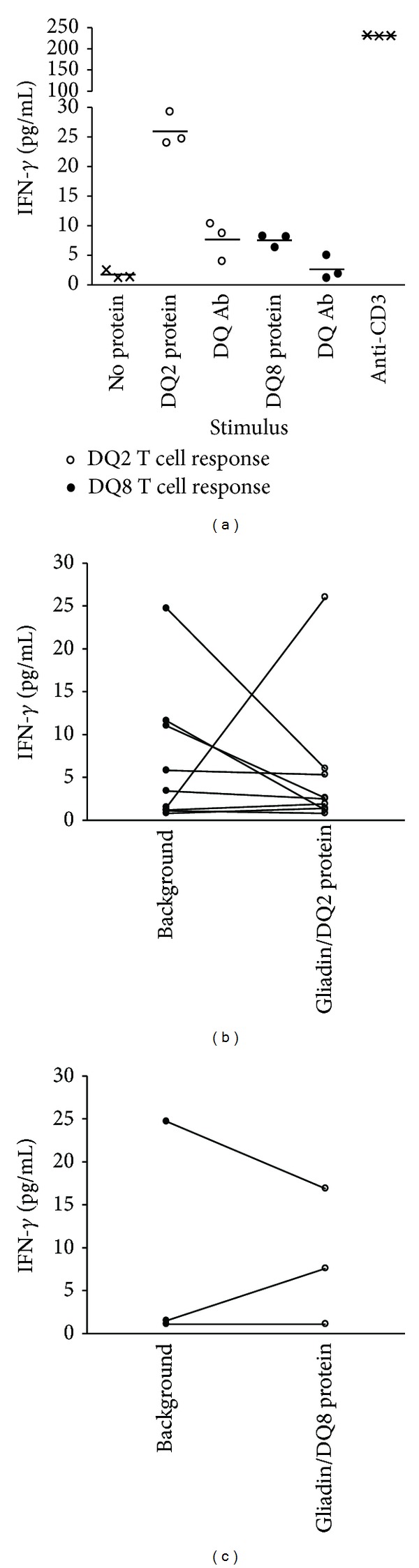
Recombinant peptide/MHC protein stimulates IFN-*γ* production from bulk unfractionated PBMCs. (a) Stimulation of PBMCs from a single subject in triplicate having both HLA-DQ8 and DQ2 alleles showing response to the recombinant proteins greater than background. The IFN-*γ* response is DQ restricted as it can be blocked with a monoclonal DQ antibody. An anti-CD3 monoclonal antibody is used to stimulate T cells as a positive control. The *α*-gliadin p1E, p9E peptide (QQYPSG**E**GSFQPSQ**E**NPQ) is present in the DQ8 recombinant protein, while *α*-I gliadin (QLQPFPQP**E**LPY) is present in the DQ2 protein. (b) Summative stimulation data from nine subjects all with HLA-DQ2 (DQA∗05:01, DQB∗02:01). (c) Data from three subjects having the HLA-DQ8 (DQA∗03:01, DQB∗03:02) allele.

T cell receptor (TCR) hybridomas, containing the TCR from a DQ2 and DQ8 restricted T cell clone responding to *α*-gliadin peptides, were created as previously described [16]. The sequences for the *αβ* TCR clones in Figure 2 were a kind gift from Ludvig Sollid. The sequences for the *αβ* TCR clones in the supplemental Figures (see supplementary material available online at http://dx.doi.org/10.1155/2014/927190) were derived from published information on gene usage [13]. Briefly, a single TCR sequence, *α* and *β* chains linked by the PTV1.2A sequence, was cloned into MSCV-based retroviral vectors carrying green fluorescent protein (GFP) (pMIGII) [17], followed by production of replication-incompetent retroviruses encoding TCR sequences. The 5KC hybridoma line lacking TCR *α* and *β* chains was used to reconstitute TCRs [18]. The transduced 5KC hybridomas were sorted by GFP expression and TCR expression was confirmed by staining with anti-mouse TCR*β* antibody (clone H57-597, BD Biosciences). Alternatively, the expression of plasmids in the pMSCVpuro retroviral vector (Clontech) was transfected to AmphoPack-293 or GP2-293 (VSV-G envelope) packaging cells (Clontech) to produce retrovirus and mouse 5KC cells were spin-infected with retroviral supernatants and cultured with puromycin. TCR expression was confirmed in isolated single clones by staining with anti-mouse CD3 (clone 145-2C11; BD Biosciences) or mouse TCR*β* antibody (clone H57-597; BD Biosciences).

### 2.4. Cytokine Stimulation Assays

Peripheral blood mononuclear cells (PBMCs) were isolated from whole blood using Ficoll-Paque and resuspended at a density of 10^6^/mL in IMDM-C media (IMDM supplemented with 5% heat inactivated human AB serum, 100 *μ*g/mL Pen-Strep, 100 *μ*M MEM NEAA, and 50 *μ*M 2-mercaptoethanol). 2 × 10^5^ PBMCs or T cell hybridomas in 200 *μ*L of media were added to a 96-well tissue culture plate coated with 1 *μ*g/well of recombinant protein and cultured at 37°C in 5% CO_2_ overnight. Secreted cytokine was measured in the supernatant by electrochemiluminescence assay (Meso Scale Discovery) for human IFN-*γ*, IL-2, and by ELISA for IL-17 (R&D Systems). PBMCs in culture without protein (background) were a negative control, while anti-CD3 stimulation (OKT3, eBioscience) was a positive control. DQ antibody SPV-L3 (Abcam, Cambridge, UK), 1a3 (Leinco, St. Louis, MO, USA), or HB-144 (ATCC, Manassas, VA, USA) was added at either 10 *μ*g/mL or at varying concentrations for blocking experiments. All study subjects had cytokine stimulation assays performed.

### 2.5. CFSE Proliferation Assay

Isolated and unfractionated PBMCs were suspended at a density of 10^6^/mL in CFSE labeling buffer (1% BSA in PBS). Cells were labeled with 1 *μ*M CFSE (eBioscience) for 10 minutes at 37°C. Labeling was quenched by adding chilled IMDM-C media at 5 times the volume at 0°C; cells were then incubated on ice for 5 minutes. Labeled cells were washed in PBS with 1% human AB serum, resuspended in media, and plated into a 24-well tissue culture plate at 10^6^ cells/well in 1 mL of media. Peptides (Genemed Synthesis Inc.) were HPLC purified (>95%), dissolved in PBS at a neutral pH, and used at a concentration of 10 *μ*M. Pentacel vaccine (Sanofi Pasteur) was added at 2 *μ*L per well as a positive control. After seven days of incubation at 37°C in 5% CO_2_, nonadherent cells were harvested and stained for FACS analysis using antibodies to CD4 (RPA-T4, BD Bioscience) and CD8 (RPA-T8, BD Bioscience). FACS analysis was done using a Becton-Dickenson FACS Caliber. Ten of the 12 study participants gave adequate numbers of PBMCs to perform CFSE proliferation assays.

### 2.6. Statistical Analysis

The percentage of CD4^+^CFSE^lo^ after proliferation to a given stimulus was compared with a paired Student's *t*-test as conditions were matched in the same subject. Response rates between *α*-gliadin and *γ*-gliadin peptides were compared with a two-sided Fisher's exact text. For all statistical tests, a two-tailed *P* value of <0.05 is considered significant. Analyses were performed using GraphPad Prism 4.0 software (La Jolla, CA).

## 3. Results

### 3.1. Subjects

Subjects with new-onset Celiac disease (*n* = 12) were recruited from the Children's Hospital Colorado Celiac Disease Center clinics. The study protocol was approved by the Institutional Review Board and written informed consent was obtained from all study participants. The Celiac subjects were young children and adolescents, with a mean age of 7.1 years, known to be TTG antibody positive and not on a gluten-free diet prior to having a small intestine biopsy. At the visit for intestinal biopsy, 11/12 (92%) patients had a biopsy and blood was collected for TTG antibody levels, HLA genotyping, and immune assays. Demographic and clinical characteristics are presented in Table 1. All of the subjects had positive TTG antibody levels and the majority 8/12 (75%) had stage 3 Marsh scores on histologic examination of a small intestine biopsy. HLA typing revealed that 9/12 (75%) subjects had the high risk HLA-DQ2 (DQA1∗05:01, DQB1∗02:01) allele and 3/12 (25%) had HLA-DQ8 (DQA1∗03:01, DQB1∗03:02) in addition to DQ2.

### 3.2. T Cell Hybridomas Respond Robustly to Recombinant Peptide/MHC Protein

We produced recombinant peptide/MHC protein to known *α*-gliadin epitopes presented by HLA-DQ2 or DQ8 (Figure 1(a)). The two recombinant proteins, *α*-I gliadin/DQ2 and deamidated *α*-gliadin p1E,p9E/DQ8, are bioactive and able to robustly stimulate T cell hybridomas created from CD4 T cells cloned from small intestine lesions of adult CD subjects [12, 13]. The responses of these T cell hybridomas, measured by secreted IL-2, are dose dependent and required minimal amounts of protein (less than 1 *μ*g/well) for stimulation (Figure 1(b), supplemental Figure 1(a)). The T cell responses are restricted to DQ2 or DQ8, depending on the class II molecule of the recombinant protein, as a DQ monoclonal antibody added in culture was able to abrogate IL-2 secretion (Figures 1(c) and 1(d) and supplemental Figures 1(b) and 1(c)).

### 3.3. Detection of IFN-*γ* Responses to Recombinant Peptide/MHC Proteins

Having recombinant *α*-gliadin/DQ proteins able to robustly stimulate T cell hybridomas, we evaluated the ability of the recombinant proteins to stimulate T cell responses from the peripheral blood of newly diagnosed CD subjects. We measured secreted cytokine responses (IFN-*γ*, IL-2, and IL-17) after culturing bulk unfractionated PBMCs in the presence of the *α*-I gliadin/DQ2 or *α*-gliadin p1E,  p9E/DQ8 recombinant protein.  Figure 2(a) depicts IFN-*γ* responses to an individual having both the DQ2 (DQA1∗05:01, DQB1∗02:01) and DQ8 (DQA1∗03:01, DQB1∗03:02) alleles, which are identical to that of the recombinant proteins. There are responses to the protein greater than that of background alone and the responses are blocked by a DQ monoclonal antibody, suggesting that the measured responses are due to reactivity to the *α*-gliadin/DQ proteins. The cumulative data from subjects having corresponding HLA alleles to that of the recombinant protein, however, failed to identify T cell reactivity above background responses (Figures 2(b) and 2(c)). The measured IL-2 and IL-17 responses were not greater than background in any of the subjects (data not shown).

### 3.4. Proliferation of CD4 T Cells to the *α*- and *γ*-Gliadin Peptides

**Figure 3:**
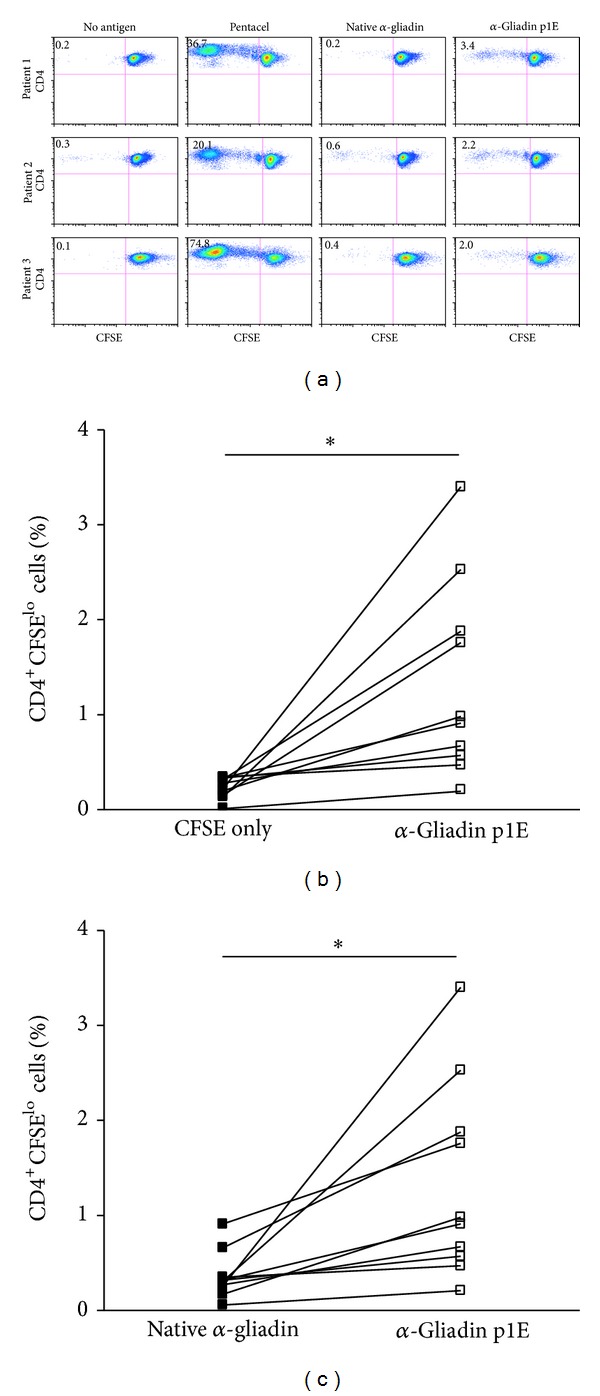
Proliferation of unfractionated PBMCs with *α*-gliadin peptides. (a) Representative data from three newly diagnosed Celiac subjects with 7-day CFSE proliferation assays. CD4 T cells proliferate in response to the *α*-gliadin p1E deamidated peptide without the in vitro addition of cytokines. (b) Summary data of proliferative responses comparing CFSE only (no antigen background) to the *α*-gliadin p1E peptide. (c) Proliferation of native *α*-gliadin to the *α*-gliadin p1E deamidated peptide. *P < 0.01 using a paired *t*-test. Pentacel (positive control) is a childhood vaccine containing immunogens directed against diphtheria, tetanus, pertussis, poliomyelitis, and *Haemophilus influenzae* type b.

**Figure 4:**
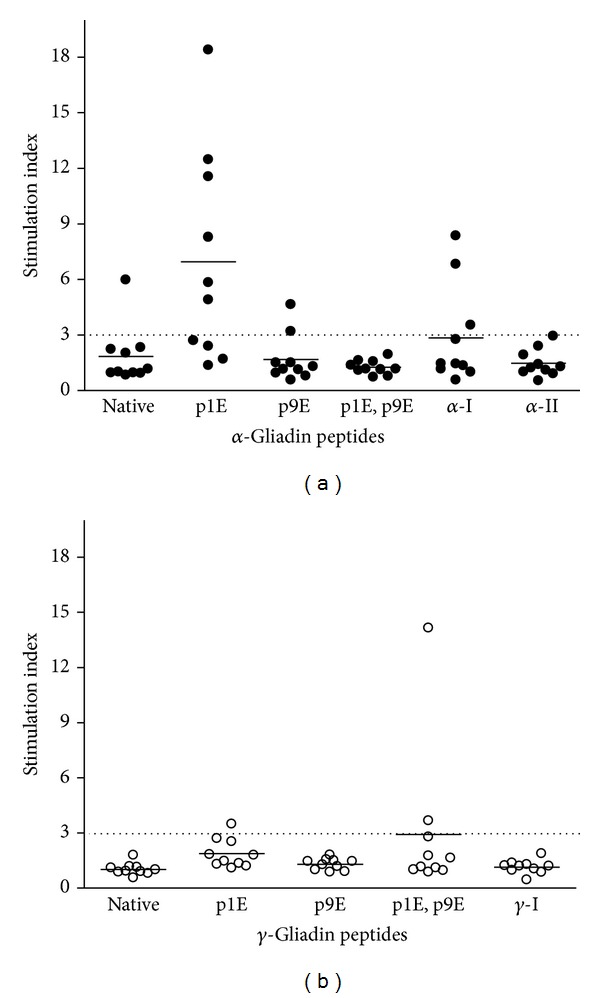
Proliferation of PBMCs from newly diagnosed Celiac patients to *α*- and *γ*-gliadin peptides.(a) Proliferative responses to *α*-gliadin and (b) *γ*-gliadin epitopes. Celiac subjects proliferate more in response to *α*-gliadin peptides compared to *γ*-gliadin, especially the peptide deamidated at pocket 1 in which 6/10 subjects responded. Overall, there are 12/60 responses to *α*-gliadin peptides compared to 3/50 for *γ*-gliadin (*P* = 0.049 with a Fisher's exact test). Dotted line is at a stimulation index (CD4^+^CFSE^lo^ cells at background/peptide condition) of 3, above which a responder is considered.

We next examined T cell responses to eleven known *α*- and *γ*-gliadin epitopes [12, 19, 20], in contrast to recombinant peptide/MHC protein, previously identified from CD patients. CD4 T cell proliferation was assessed from the peripheral blood of newly diagnosed CD subjects.  Figure 3 shows the proliferation results after bulk unfractionated PBMCs were labeled with CFSE and cultured for 7 days in the presence of a single gliadin peptide without the addition of any in vitro stimulus, that is, no IL-2, anti-CD3, or anti-CD28. Of the individuals having CFSE proliferation assays performed, there were robust responses to the *α*-gliadin_228-240_ peptide (SGQGSFQPSQQNP), especially with a deamidated glutamate residue at the pocket 1 position. In all of the subjects, the single deamidated peptide (SG**E**GSFQPSQQNP) resulted in significantly more proliferation as measured by CD4^+^CFSE^lo^ cells compared to background (no antigen in culture) and the native *α*-gliadin peptide (Figures 3(b) and 3(c)). Interestingly, none of the subject's PBMC proliferated in response to the double deamidated *α*-gliadin peptide (*α*-gliadin p1E, p9E) and few responded (3/10) to the *α*-I-gliadin peptide, which are the peptides in the DQ8 and DQ2 recombinant protein, respectively (Table 2). Evaluating proliferative responses with a stimulation index (CD4^+^CFSE^lo^ condition/CD4^+^CFSE^lo^ background) greater than 3 revealed 12/60 (20%) responses to *α*-gliadin peptides compared to 3/50 (6%) of *γ*-gliadin peptides (*P* = 0.049). Overall, there were more proliferative responses to *α*-gliadin peptides compared to *γ*-gliadin in young children with newly diagnosed CD (Figure 4). In those children responding to two or more peptides, all three were HLA-DQ2/2 homozygotes (Table 3). Interestingly, the HLA-DQ2/2 children responded to epitopes which have been previously reported in the literature to be DQ8 restricted, suggesting that certain *α*-gliadin peptides may be presented by either DQ8 or DQ2, particularly after selective deamidation.

## 4. Discussion

In the present study, we investigated peripheral T cell responses from young children with newly diagnosed CD prior to treatment with a gluten-free diet for two purposes: (1) develop a consistent and rapid assay to monitor peripheral T cell responses and (2) assess T cell responses to a panel of *α*- and *γ*-gliadin epitopes. To develop a consistent and rapid assay using limited PBMCs, *α*-gliadin/DQ2 and DQ8 recombinant protein constructs were produced which have the ability to robustly stimulate six different T cell clones derived from adult CD patients, suggesting the utility of this approach with polyclonal T cells from the peripheral blood. However, stimulation of PBMCs from young untreated children with CD did not uniformly elicit T cell responses. There are several possible reasons for the inability to detect reactivity with these protein constructs including the possibility that CD donors lack T cells that recognize a single DQ2 or DQ8 *α*-gliadin epitope. Interestingly, the double deamidated *α*-gliadin epitope covalently linked to recombinant DQ8 protein failed to elicit proliferative T cell responses in our study population, while three individuals with proliferative responses to *α*-I gliadin did not produce IFN-*γ* above background levels when stimulated with the recombinant *α*-I gliadin/DQ2 protein. As these epitopes were identified from adult CD patients, our data supports the heterogeneity of T cell responses in CD and that children may respond to different epitopes than adults [21]. Second, the donor's T cells may already be maximally stimulated as our patient population had not yet started a gluten-free diet. This is in agreement with studies reporting the need for a short-term gluten challenge in adult CD patients already on a gluten-free diet to elicit robust peripheral T cell responses [8, 9]. However, peripheral T cell responses can exist at disease onset and disappear with a gluten-free diet. Finally, it is possible that measuring secreted IFN-*γ* may not be the best way to detect responses, and other methods for detecting antigen specific T cell responses to gliadin may be necessary, such as using gliadin/DQ2 fluorescent tetramers [22].

In addition to evaluating peptide/MHC recombinant protein to elicit T cell responses, we utilized proliferation assays with bulk unfractionated PBMCs to assess peptide reactivity. Stimulation of PBMCs from newly diagnosed and untreated CD children is better detected using proliferation assays rather than measurement of secreted cytokine. In our panel of DQ2 and DQ8 restricted antigens, we found that 6/10 children responded to at least one of the peptides tested, and the remainder did not respond to any tested peptide. It is notable that a peptide traditionally considered to be a DQ8 epitope, *α*-gliadin p1E ^228^SG**E**GSFQPSQQNP^240^, showed the greatest ability to stimulate T cells in 60% of children, even though only two children expressed the HLA-DQ8 allele (all of the children had at least one DQ2 allele). Furthermore, there were three subjects that had responses to two or more peptides and all three were DQ2/DQ2 homozygotes (Table 3). The peptide binding grooves of HLA-DQ2 and DQ8 share structural similarity with both molecules capable of anchoring peptides with acidic side chains (glutamic and aspartic acid) at pockets 1 and 9 [23]. With tissue transglutaminase present to deamidate gliadin peptides, it is plausible to hypothesize that certain deamidated epitopes have the potential to be presented by both the DQ2 and DQ8 molecules [24, 25]. There is precedence for this concept as both HLA-DR1 and DR4 class II molecules are capable of presenting the hemagglutinin peptide, HA_306–318_, to T cells [26, 27].

In summary, T cell reactivity in young children with newly diagnosed and untreated CD is heterogeneous but favors reactivity to *α*-gliadin epitopes more than *γ*-gliadin. It is unlikely that a single gliadin epitope will elicit T cell responses in all individuals and a short-term gluten challenge may be necessary to detect ample T cell reactivity in the peripheral circulation. However, it is possible to detect T cell responses without a gluten challenge or in vitro stimulus other than antigen, when measuring proliferative responses. A more comprehensive screening of gliadin epitopes in young children with newly diagnosed CD is necessary to identify peripheral blood T cell reactivity, followed by repeat assessments over time to correlate responses to disease activity and treatment with a gluten-free diet. Monitoring peripheral T cell responses to gliadin epitopes present at disease onset, which can disappear with a gluten-free diet, has clinical utility in identifying cases of refractory CD or in assessing the effectiveness of emerging therapies for CD treatment.

## Supplementary Material

The Supplementary Material contains one figure depicting the response of four separate T cell clones responding to the a-gliadin/DQ8 recombinant protein construct.

## Figures and Tables

**Table 1:** Clinical characteristics, TTG antibody levels, histology, and HLA genotype of study participants.

Case	Age (yrs)	Sex	TTG Ab level*	Histology marsh score	HLA DQ and DR alleles					
					DRB1	DQA1	DQB1	DRB2	DQA2	DQB2
1	4	F	0.145	2	0404	0301	0302	0301	0501	0201
2	5	F	0.130	0	0403	0301	0302	0301	0501	0201
3	5	M	0.064	1	0301	0501	0201	1602	0501	0301
4	5	M	0.178	3b	0301	0501	0201	0301	0501	0201
5	4	F	1.127	3c	0301	0501	0201	0801	0401	0402
6	7	F	0.877	No biopsy	0701	0201	0202	1104	0501	0301
7	7	F	0.608	3c	0301	0501	0201	1301	0103	0603
8	7	M	0.755	3b	0301	0501	0201	0701	0201	0202
9	7	F	0.461	3b	0301	0501	0201	1501	0102	0602
10	12	F	0.624	3b	0701	0201	0202	1101	0501	0301
11	13	M	0.511	3b	0403	0301	0302	0701	0201	0202
12	9	F	0.169	3b	0301	0501	0201	0301	0501	0201

*TTG Ab ≥ 0.05 is elevated.

**Table 2:** Proliferative responses to  *α*- and *γ*-gliadin epitopes.

Epitope	Amino acid sequence*	Proliferation response**
Native *α*-gliadin	SGQGSFQPSQQNP	1/10 (10%)
*α*-Gliadin p1E	SG**E** GSFQPSQQNP	6/10 (60%)
*α*-Gliadin p9E	SGQGSFQPSQ **E**NP	2/10 (20%)
*α*-Gliadin p1E, p9E	SG**E** GSFQPSQ **E**NP	0/10 (0%)
*α*-I-Gliadin	QLQPFPQP **E** LPY	3/10 (30%)
*α*-II-Gliadin	PQP **E** LPYPQPQL	0/10 (0%)
Native *γ*-gliadin	FPQQPQQPYPQQPQQ	0/10 (0%)
*γ*-Gliadin p1E	FP**E** QPQQPYPQQPQQ	1/10 (10%)
*γ*-Gliadin p9E	FPQQPQQPYP **E**QPQQ	0/10 (0%)
*γ*-Gliadin p1E, p9E	FP**E** QPQQPYP **E**QPQQ	2/10 (20%)
*γ*-I-Gliadin	PEQPQQSFP **E** QERP	0/10 (0%)

*Glutamic acid (E) residues in bold are formed by tissue transglutaminase mediated deamidation. Underlined residues form the MHC class II peptide binding register.

**A stimulation index ≥ 3 is considered a response.

**Table 3:** Overview of the T cell responses to tested DQ2 and DQ8 gliadin epitopes.

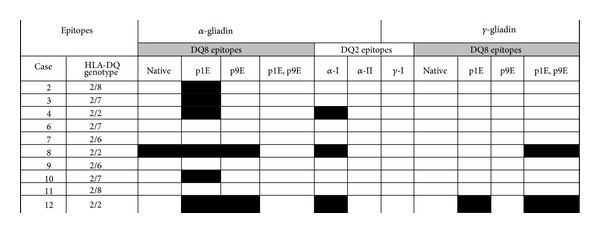

Peripheral T cell responses as measured by CD4^+^CFSE^lo^ proliferated cells for each individual with correlation to HLA-DQ genotype. Black boxes represent a response to the peptide with the SI ≥ 3. Gliadin epitopes are denoted as previously reported in the literature to be presented by HLA-DQ2 (*α*-I gliadin_57–68_ QLQPFPQPELPY, *α*-II gliadin_62–73_ PQPELPYPQPQL, and *γ*-1 gliadin_139–152_ PEQPQQSFPEQERP) or HLA-DQ8 (*α*-gliadin_228–240_ SGQGSFQPSQQNP and *γ*-gliadin_65–79_ FPQQPQQPYPQQPQQ with and without deamidation at p1 and p9). Three of the new-onset CD children responding to two or more peptides have the DQ2/2 genotype.
