# Supplementary material for: Exploring T Cell Reactivity to Gliadin in Young Children with Newly Diagnosed Celiac Disease
Source: Autoimmune Dis. 2014 Mar 3;2014:927190. doi: 10.1155/2014/927190 (PMC3958769; doi:10.1155/2014/927190)
Supplement: Supplementary file 1 — The Supplementary Material contains one figure depicting the response of four separate T cell clones responding to the a-gliadin/DQ8 recombinant protein construct. [file 927190.f1.pdf]

Supplementary Data

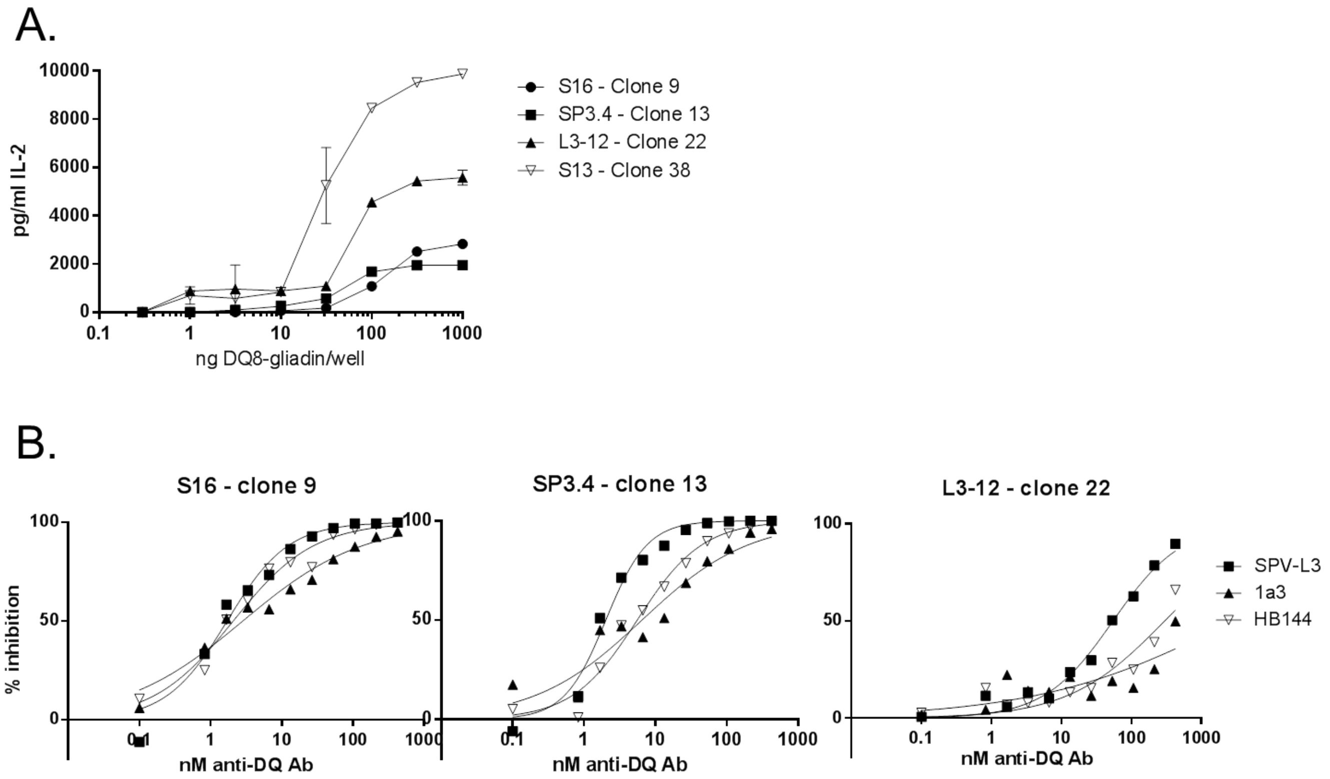

C.

IC50 (nM)	Anti-DQ antibody		
	SPV	1a3	HB144
S16 - Clone 9	1.5	2.4	2.1
SP3.4 - Clone 13	21.4	6.8	5.6
L3-12 - Clone 22	54.5	>425	296.7

Supplemental Figure 1. Gliadin/DQ8 recombinant protein stimulates different DQ8 restricted T cell clones. (A) Four HLA-DQ8 restricted T cell clones produce robust amounts of IL-2 in response to the deamidated α-gliadin/DQ8 recombinant protein. (B) The T cell response can be blocked with different commercial DQ monoclonal antibodies with the (C) IC_{50} values depicted for each T cell clone and DQ antibody.
